# Squamous Cell Carcinoma of the Lung in McCune-Albright Syndrome

**DOI:** 10.7759/cureus.14159

**Published:** 2021-03-28

**Authors:** Vinai Y Reddy, Erika Tvedten, Muthanna Louis

**Affiliations:** 1 Internal Medicine, Michigan State College of Osteopathic Medicine, Detroit, USA; 2 Internal Medicine, Detroit Medical Center (DMC) Harper Hospital, Detroit, USA

**Keywords:** mccune albright syndrome, gnas mutation, squamous cell carcinoma, hypercalcemia of malignancy, fibrous dysplasia

## Abstract

McCune Albright Syndrome (MAS) is caused by a mutation in the GNAS gene that results in multiple endocrinopathies such as Cushing syndrome, acromegaly, hyperthyroidism, and precocious puberty. Despite the presence of pleiotropy coupled with a GNAS gene mutation, malignancy is a rare occurrence in MAS. There is minimal literature showcasing squamous cell carcinoma (SCC) of the lung in patients with MAS. Here, we report a case of altered mental status and hypercalcemia in a 72-year-old female with a past medical history of fibrous dysplasia. On examination there was an expansive lesion of the left hemipelvis that had been present since birth; it was determined to be a characteristic of polyostotic fibrous dysplasia seen in MAS. Lab results revealed an elevated parathyroid hormone-related protein (PTHrP) and a chest X-ray (CXR) displayed masses in the right upper lobe. Computed tomography (CT)-guided lung biopsy confirmed the masses to be SCC of the lung. This case primarily highlights the importance of investigating other malignancies found in patients with MAS. Current literature shows that the GNAS mutation is an oncogenic driver in many tumor types such as pancreatic adenocarcinoma, adrenocortical carcinoma, and thyroid carcinoma; however, current data are limited on the role of GNAS mutations in SCC of the lung. In addition, it is important to investigate malignant causes of hypercalcemia in patients with MAS. The clinical features and treatments of MAS can lead to hypercalcemia and hypophosphatemia and thus mask a malignancy. Clinicians must be wary of a masked malignancy, as it could delay treatment and negatively impact overall outcomes for patients with MAS.

## Introduction

McCune Albright Syndrome (MAS) is a rare disease with the classic triad of polyostotic fibrous dysplasia of bone, unilateral café-au-lait spots, and at least one endocrinopathy such as precocious puberty, hyperthyroidism, or Cushing syndrome. The estimated prevalence of MAS ranges from 1/100,000 to 1/1,000,000 [1]. Patients with MAS exhibit somatic mosaicism of the GNAS gene, more specifically mutations in the Gs alpha subunit of the cyclic adenosine monophosphate (cAMP) regulating protein [2]. Patients with MAS have a gain-of-function mutation in the GNAS gene causing adenylate cyclase to be constitutively activated thus ultimately creating an overproduction of hormones and intracellular signals [3].

GNAS mutations have been shown to play an oncogenic role in the pathogenesis of thyroid, pituitary, and pancreatic carcinomas [4-5]. Despite the widespread effects of GNAS, malignancy associated with MAS is a rare occurrence. Malignant transformation of fibrous dysplasia has been shown to be the most common malignancy and is present in under 1% of individuals with MAS [2]. The literature is very limited on the prevalence of squamous cell carcinoma in patients with MAS. This case report aims to highlight a rare case of squamous cell carcinoma of the lung in MAS and illustrates that the clinical features of MAS can mask potential malignancy.

## Case presentation

We report a case of a 72-year-old African American female with a past medical history of fibrous dysplasia, deep vein thrombosis (DVT), gastroesophageal reflux disease (GERD), hypertension (HTN), and unknown social history of cigarette smoking who presented with altered mental status (AMS). The patient initially presented to the emergency department (ED) alert and oriented (AO) x1, indicating she could only state her name and recognize her significant others. The patient's mental status soon improved to AOx3 as she was oriented to person place and time. She was, however, unaware of why she was brought to the hospital. It was noted that the patient’s mentation was waxing and waning although she had no history of dementia prior to admission. Heart, lung, and abdominal exam provided no abnormalities. Neurological exam revealed cranial nerves 2 through 12 were intact with normal motor and sensation. The lower extremity exam showed deformity in the legs, swelling with palpable pulses, and no associated cyanosis. The patient stated that the expansive lesion of her left hemipelvis had been present lifelong and, therefore, it was determined to be the characteristic of polyostotic fibrous dysplasia seen in McCune Albright syndrome.

The patient’s labs showed that her serum calcium was 15.3 mg/dL (corrected calcium was 16.2 mg/dL). Albumin level was 3.0 gm/dL. Vitamin D 25-hydroxy was low at 15.6 ng/mL and calcitriol was 31 pg/ml. The parathyroid hormone (PTH) level was low at 11 pg/mL. The parathyroid hormone-related protein (PTHrP) level was high at 61 pg/ml. Thyroid-stimulating hormone (TSH) was 1.02 mcIU/mL. The serum-free light chain (FLC) ratio was normal and serum protein electrophoresis (SPEP) showed no monoclonal bands. Computed tomography (CT) and magnetic resonance imaging (MRI) of the head were unremarkable. A CXR revealed a stable right upper lobe mass, and the patient was discharged for an outpatient biopsy. The patient was lost to follow-up for three weeks before presenting to the ED again.

**Figure 1:**
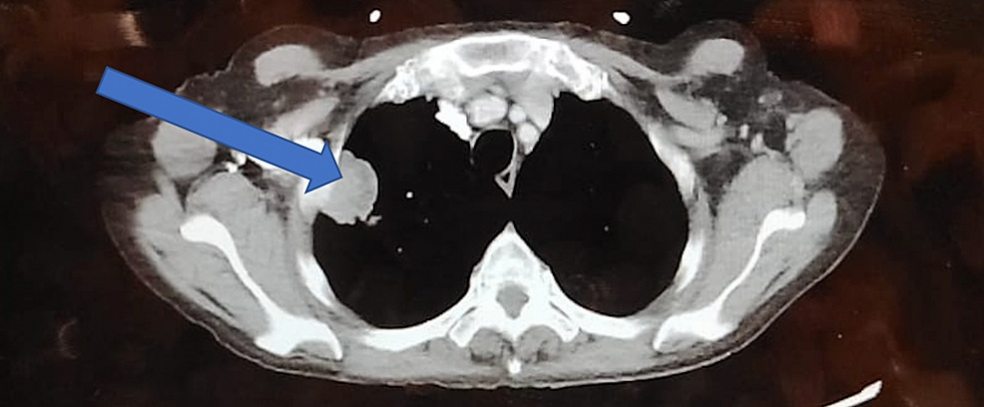
CT scan illustrating malignancy (squamous cell carcinoma) in the right upper lobe

A CT of the chest abdomen and pelvis showed multiple soft tissue attenuating masses in the right upper lobe lung originating from the lateral chest wall. Of these masses, the largest measured 3.4 cm in dimension (Figure 1). In addition, there was a mass with soft-tissue attenuation within the right upper lobe which measured 2.0 cm in diameter. Additionally, multiple abnormalities consistent with the patient’s history of Mazabraud syndrome were identified on the scan.

The presence of lung lesions and a negative hypercalcemia workup except for an elevated PTHrP was concerning for a paraneoplastic syndrome. Workup for malignancy was begun, and the patient was started on zoledronic acid 4 mg and calcitonin 300 units.

The patient underwent a CT-guided lung nodule biopsy via interventional radiology (IR) confirming the diagnosis of squamous cell carcinoma (SCC) of the lung. A positron emission tomography (PET) scan showed peripheral hypermetabolic activity of the neoplasm in the right upper lobe. The soft tissue density, thought to be the lymph node, at the left cardio-phrenic space also showed increased metabolic activity, indicating likely metastasis. Focal areas of increased metabolic activity in the T10 and T11 vertebral bodies were also presumed to be metastatic lesions.

The patient had subsequent comorbidities that arose and became apparent during her stay: anemia of chronic disease, acute kidney injury (AKI), hypoglycemia secondary to poor nutrition, shortness of breath (SOB) secondary to volume overload, thrombocytosis, persistent leukocytosis, and acute urinary tract infection (UTI) that progressed to left-sided pyelonephritis.

The patient’s overall performance status rapidly declined from the time of admission, and she developed profound weakness, debility, and deconditioning secondary to her current diagnosis. She developed pressure ulcers secondary to her lack of mobility with activity. With the patient’s poor prognosis, it was determined she would likely be unable to tolerate chemotherapy. Palliative care was consulted, and hospice was considered. The patient was discharged to subacute rehab (SAR).

## Discussion

Patients with MAS have a GNAS mutation, which has been known to play a role in the pathogenesis of many endocrine cancers such as pancreatic adenocarcinoma, gastric carcinoma, and thyroid carcinoma [3]. Patients with MAS have mosaicism of the GNAS mutation, thus the mutation is only present in some of the body’s cells but not others. Literature shows that cells with the mutation can only co-exist with the normal variant of cells, and the extent of the disease is based on the migration and proliferation of the cells with the mutation [6]. If the mutation in GNAS is ubiquitous throughout the body, the mutation would be lethal and lead to loss of the zygote in utero. The presence of the GNAS mutation can lead to a significant tissue burden in several organs, but interestingly, the prevalence of malignancy in MAS is extremely rare. It is postulated this may be due to the weak cumulative oncogenic effect of GNAS mutations; the ratio of cells with the GNAS mutation to the normal variant in cells is extremely small thus suggesting the interaction of external oncogenic pathways may be necessary for cancer pathogenesis [4]. The weak cumulative oncogenic effect of the GNAS mutation can also be a potential explanation for why the patient presented with malignancy at the age of 72 and not earlier on in her life.

Squamous cell carcinoma of the lung can be caused by a variety of environmental factors such as smoking, recurrent inflammation from pneumonia, radiation, and exposure to irritants such as asbestos or arsenic [4]. Tobacco is the highest environmental risk factor for squamous cell carcinoma of the lung. Therefore, with the patient's unknown smoking history, it can be postulated that her potential use of tobacco may have played a role in developing squamous cell carcinoma of the lung. Smoking may have been the necessary extrinsic factor that amplified the initially weak cumulative effect of the GNAS mutation to develop into squamous cell carcinoma of the lung. Further research comparing individuals who smoke and have the GNAS mutation to individuals who are just smokers can help quantify the role smoking has on patients with a GNAS mutation. On the other hand, some researchers believe that the GNAS mutation in MAS could have a protective effect, as the overall incidence of malignancy in MAS is very low [3]. Ultimately, more data is needed to determine if patients with MAS inherit protective traits that may allow for apoptosis or quiescence of tumor-bearing cells [4].

Since our patient with a past medical history of MAS presented to the ED with hypercalcemia and AMS, it was imperative to determine the etiology of the hypercalcemia to determine the potential of an underlying malignancy. Hyperparathyroidism with phosphate wasting is a common finding in patients with MAS, which can also present with an elevated calcium level secondary to the elevated parathyroid level. In addition, patients with MAS classically present with fibrous dysplasia of bone (FD). Although FD does not primarily cause hypercalcemia, calcium levels can be elevated through immobilization secondary to the debilitation of FD. The medications used for the treatment of FD can also play a role in a patient’s serum calcium levels. Patients with FD have overexpression of RANK ligand (RANKL), a cell surface marker used in osteoclastogenesis causing an increased bone degradation and remodeling [7]. Denosumab is a monoclonal antibody against RANKL used primarily for osteoporosis but has been shown to be a promising treatment in patients with FD, especially when bisphosphonates have been ineffective [8]. Despite the promising nature of using denosumab for FD, patients taking denosumab may experience hypocalcemia and hypophosphatemia while on treatment, and abrupt discontinuation when entering the hospital can cause life-threatening hypercalcemia [9]. These causes of hypercalcemia revolve around the pathogenesis and treatments of MAS, however, distinguishing hypercalcemia due to symptoms pertinent to the disease versus hypercalcemia from malignancy is imperative to the patient’s prognosis.

The patient presenting with hypercalcemia and altered mental status was worked up with CXR and CT-guided lung biopsy thus determining that the hypercalcemia was indeed due to malignancy: SCC of the lung. Hypercalcemia due to malignancy is caused by three potential factors, which include tumor secretion of PTHrP, osteolytic metastases with an elevation of cytokines and osteoclast activating factors, and tumor secretion of calcitriol (1,25- dihydroxyvitamin D) [10]. Our patient exhibited an elevated PTHrP. PTHrP is regulated by the Hedgehog pathway and Gli transcriptional factors, a similar process thought to be present in carcinogenesis. PTHrP stimulates a hyperactive pathway of osteoclast resorption leading to bone turnover and hypercalcemia [11]. PTHrP can also cause hypophosphatemia. Since PTHrP is structurally similar to PTH, there is an increase in phosphate excretion and calcium reabsorption in the renal tubules [12]. Hypophosphatemia is also found in patients with MAS due to the osteogenic precursors of fibrous dysplasia producing fibroblast growth factor 23 (FGF-23) [13]. FGF-23 inhibits renal tubular phosphate absorption leading to phosphate wasting and hypophosphatemia in patients with MAS. Therefore, similar to PTHrP, patients with MAS can have similar electrolyte abnormalities, hypercalcemia, and hypophosphatemia, which can mask malignancy.

## Conclusions

Overall, in this case report, we highlight the importance of investigating the oncogenic effects of GNAS mutations and their potential for squamous cell carcinoma. We also want to be cognizant of patients with rare disorders such as McCune Albright Syndrome who present with hypercalcemia. An early workup for malignancy can aid in faster diagnosis and improved treatment outcomes.
